# Ectopic adrenal nodular hyperplasia mimicking right upper polar renal cell carcinoma: a case report and literature review

**DOI:** 10.3389/fonc.2026.1731776

**Published:** 2026-02-19

**Authors:** Shuxin Li, Yongliang Qu, Yongshuai Huang, Si Liu

**Affiliations:** 1 Department of Urology, The First Hospital of Jilin University, Changchun, China; 2 Organ Transplant Center, The First Hospital of Jilin University, Changchun, China; 3 Jinzhou Medical University, Jinzhou, China

**Keywords:** case report, ectopic adrenal gland, painless hematuria, partial nephrectomy, renal cell carcinoma

## Abstract

Ectopic adrenal tissue refers to adrenal tissue appearing in an abnormal anatomical location, typically originating from residual adrenal tissue or ectopic structures during embryonic development. As a rare congenital anomaly, its atypical anatomical position and diverse clinical manifestations often pose diagnostic challenges. This report describes a 46-year-old male patient who presented with intermittent hematuria for two weeks. Enhanced CT of the kidneys revealed a protruding nodule at the margin of the right renal pole, measuring approximately 1.6 cm and showing relatively uniform enhancement, suggesting a hypo vascular mass lesion. Enhanced MRI of both kidneys showed a nodule at the right renal pole, consistent with a mass due to insufficient blood supply. Based on imaging findings and clinical symptoms, a preliminary diagnosis of right renal carcinoma (RCC) was made, leading to a laparoscopic partial nephrectomy. Postoperative pathology confirmed the lesion as ectopic adrenal tissue. This case highlights that ectopic adrenal tissue within the kidney may mimic renal cell carcinoma in both clinical presentation and imaging characteristics, underscoring the need for enhanced differential diagnosis between these two conditions in clinical practice.

## Introduction

Ectopic adrenal glands refer to adrenal tissue appearing outside its normal anatomical location, typically situated beyond the renal region (1). Such tissue may consist solely of the adrenal cortex or represent complete glandular structures (1). Its occurrence is related to the close association between the adrenal and gonadal primordia during embryonic development, which results in residual tissue remaining in abnormal locations during migration (2, 3). Consequently, ectopic adrenal tissue is also referred to as an adrenal remnant or ectopic adrenal cortical tissue, representing a location anomaly rather than a functional defect (4, 5). This condition is more prevalent in male children and relatively uncommon in females (4, 6). The incidence of ectopic adrenal tissue in adults is less than 1% (7). The most common sites are the genitourinary system and pelvis, with other rare locations including the retroperitoneum, hepatic capsule, gallbladder, and nervous system (1, 8–12). The distribution of ectopic adrenal glands exhibits gender differences, with males more frequently presenting them in reproductive structures such as the spermatic cord and epididymis. At the same time, females are more commonly affected, with them often being harbored within the ovaries and fallopian tubes (6, 12–14). Most ectopic adrenal tissue is asymptomatic and is discovered incidentally during surgery or pathological examination (11). In rare cases, this tissue may develop into functional tumors, causing abnormal hormone secretion (2, 8). For example, ectopic adrenal cortical adenomas can lead to Cushing’s syndrome, presenting weight gain, hypertension, and hypercortisolemia (2, 8, 15). Pheochromocytomas originating from ectopic adrenal medulla may cause symptoms like paroxysmal hypertension and palpitations, though this is infrequent (16, 17). Once ectopic adrenal tissue is identified, surgical resection is recommended regardless of clinical symptoms (13, 18). This is due to the tissue’s potential for malignant transformation and its capacity to disrupt endocrine function (15). Furthermore, ectopic adrenal tissue is frequently misdiagnosed as malignant tumors such as renal cell carcinoma or hepatocellular carcinoma on imaging studies (5, 19). Early resection facilitates definitive diagnosis and prevents unnecessary overtreatment. If suspicious adrenal tissue is incidentally discovered during abdominal or pelvic surgery, it should be resected and sent for pathological examination (18, 20). If pathology confirms ectopic adrenal tissue, no further treatment is typically required, though regular follow-up is recommended.

This diagnostic challenge is particularly pronounced when ectopic adrenal tissue is located within the renal parenchyma, where its radiological features can closely mimic those of RCC. The presence of cystic change or pseudocyst formation within intrarenal ectopic adrenal tissue, though rare, further complicates the imaging interpretation and increases the risk of preoperative misdiagnosis. Moreover, when such lesions present symptoms typical of RCC, such as painless hematuria, the clinical suspicion for malignancy is heightened, often leading to surgical intervention. This case report aims to present a rare instance of intrarenal ectopic adrenal tissue with pseudocyst formation that clinically and radiologically mimicked a right upper polar renal cell carcinoma, resulting in a laparoscopic partial nephrectomy. We discuss the preoperative diagnostic dilemmas, highlight the overlapping features with RCC, and review the relevant literature to enhance awareness of this benign mimic among clinicians and radiologists. We hope to contribute to more accurate preoperative assessments and to inform the management of similar cases in the future.

## Case presentation

**Figure 1:**
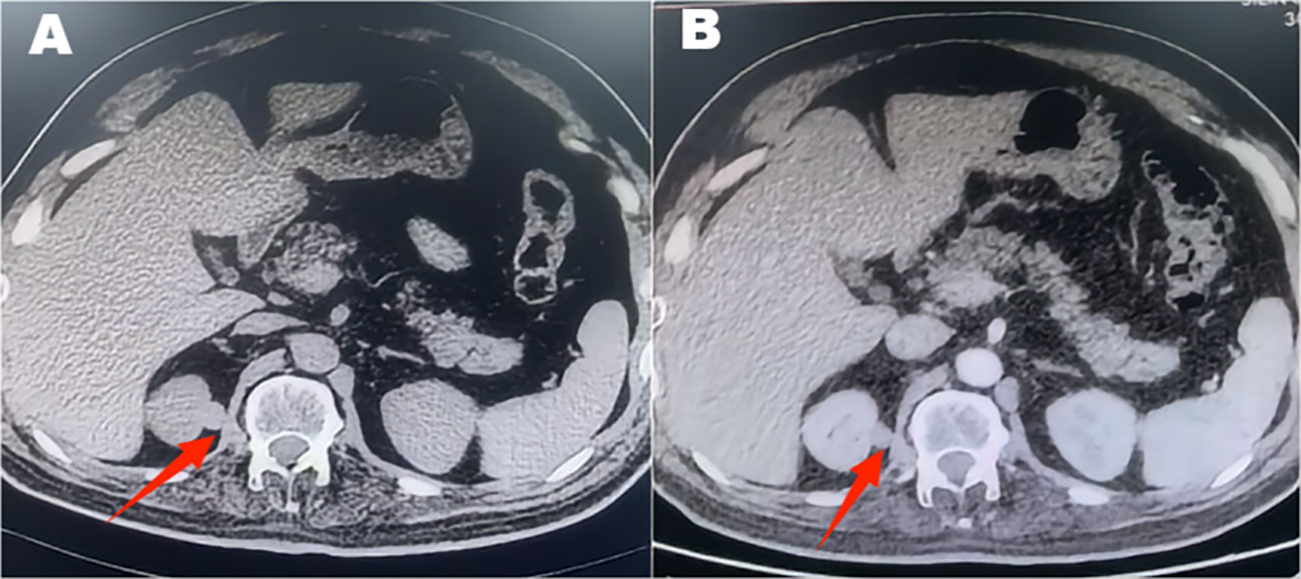
Contrast-enhanced CT demonstrates a well-defined, exophytic, hypodense nodule at the upper pole of the right kidney, showing minimal enhancement.

**Figure 2:**
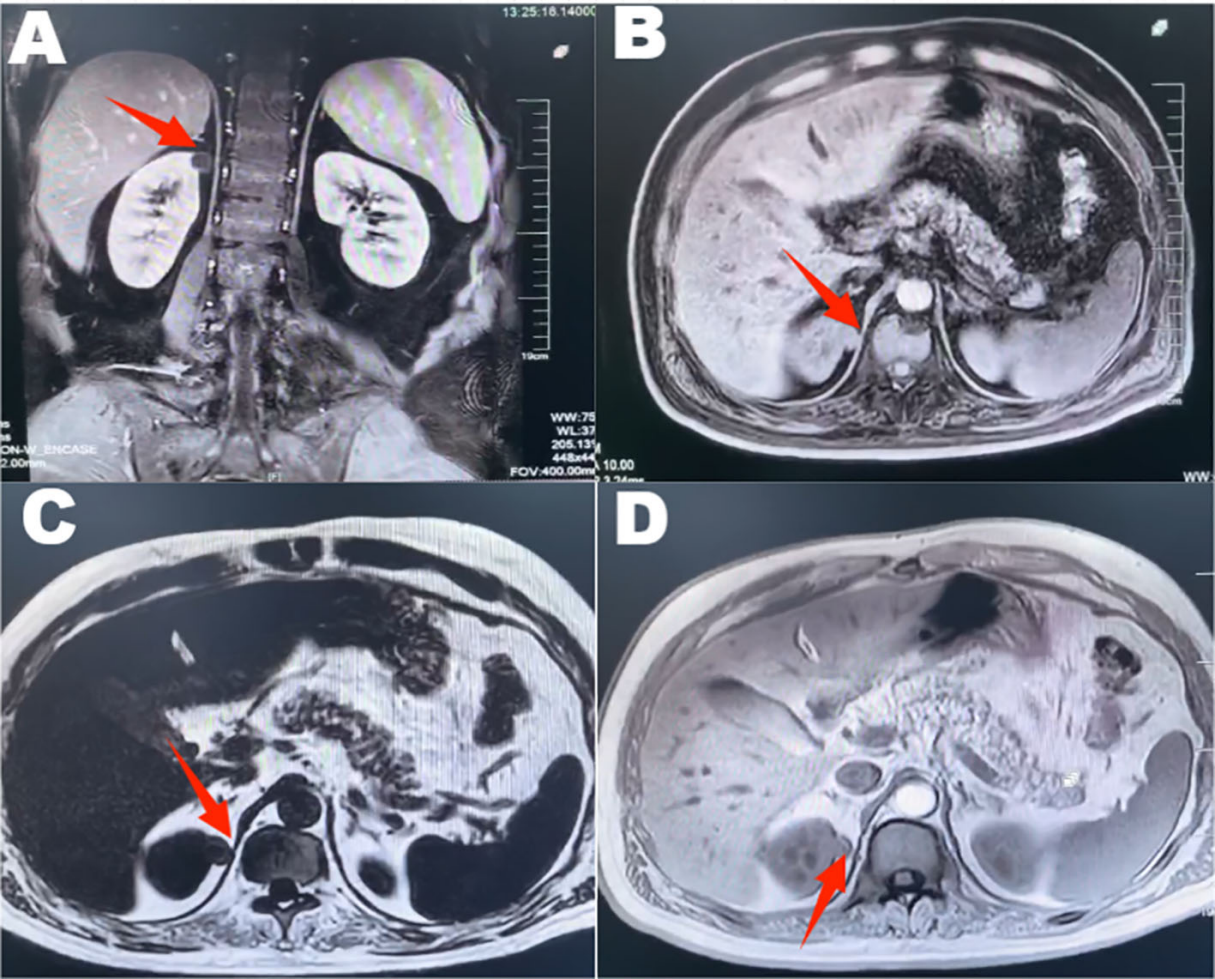
MRI reveals a protruding nodular lesion measuring approximately 1.6 cm at the margin of the right renal upper pole, exhibiting mildly prolonged T1 and T2 signal intensity. The lesion demonstrates low signal intensity on diffusion-weighted imaging and shows no significant enhancement on contrast-enhanced scans. This indicates the presence of an occupying lesion in the right upper renal pole, with associated hypoperfusion.

**Figure 3:**
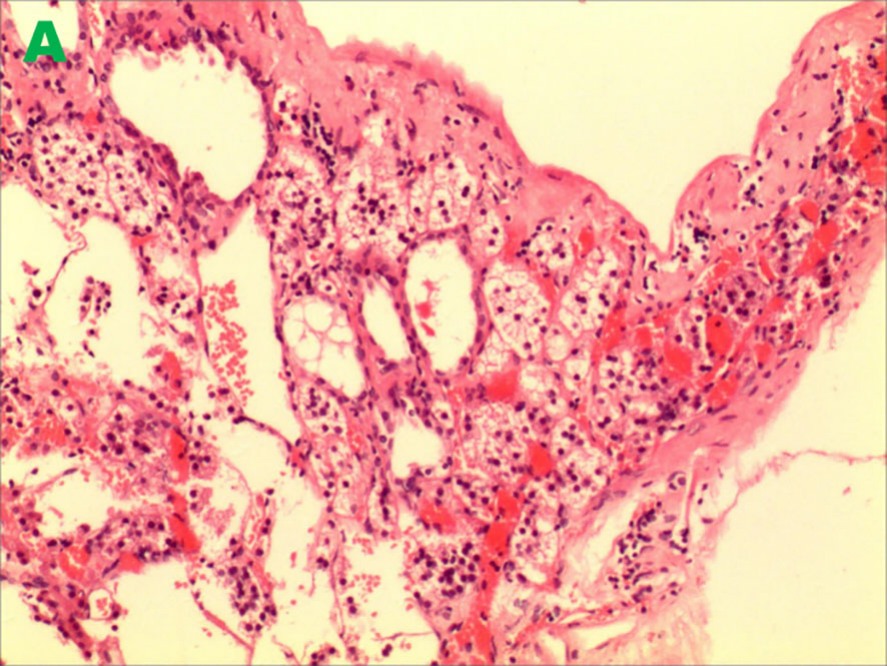
Ectopic adrenal tissue within the kidney forming a pseudocyst.

**Figure 4:**
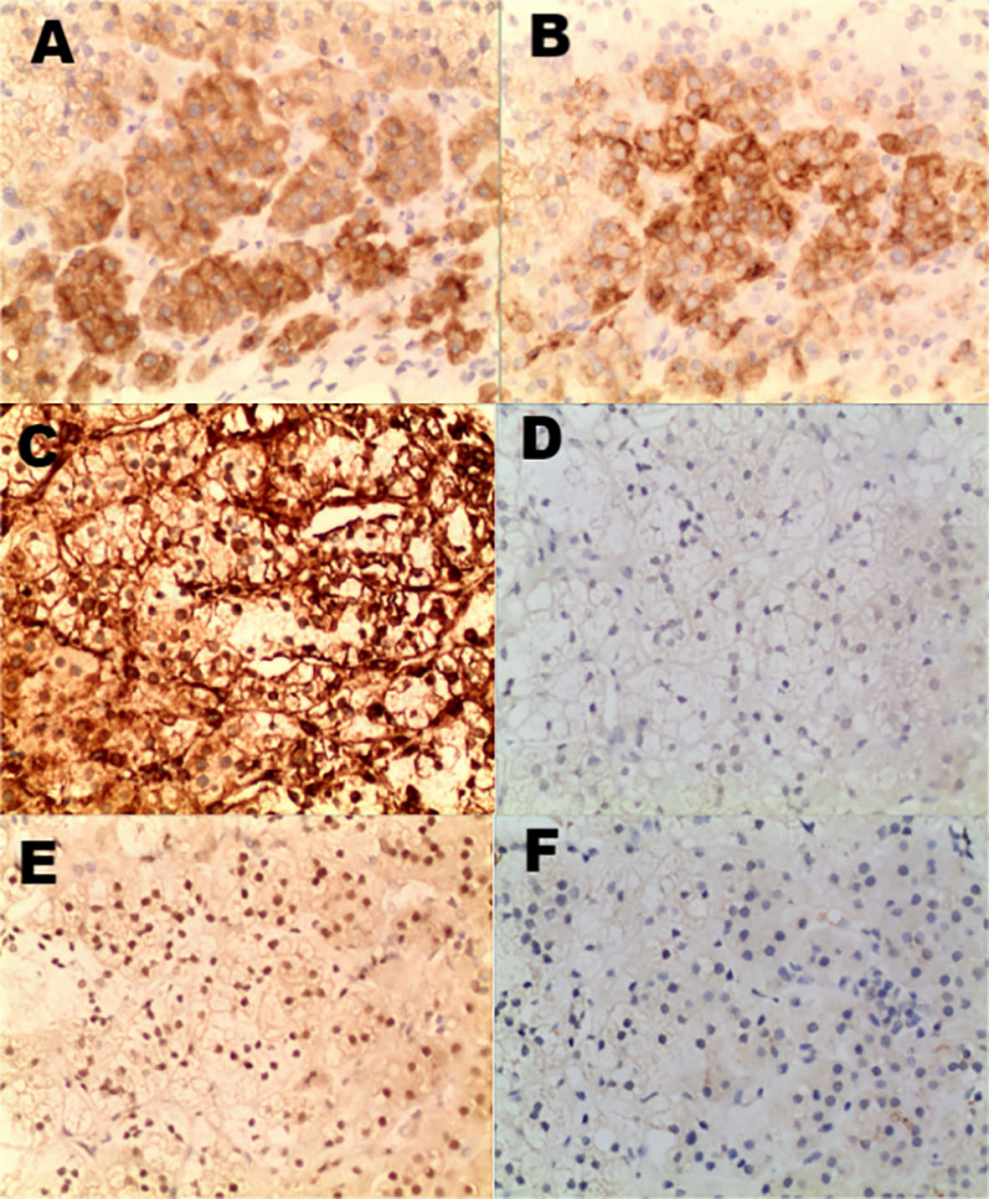
Immunohistochemical staining shows strong positivity for Inhibin **(A)**, Synaptophysin **(B)** and Vimentin **(C)**; demonstrates nuclear positivity for TFE3 **(E)**; and is negative for renal markers including PAX-8 **(D)** and CD10 **(F)**.

A 46-year-old male patient presented with intermittent gross hematuria for 2 weeks. The hematuria was characterized as terminal hematuria, appearing pale red. The patient had no significant past medical history, no family history of similar conditions, and was otherwise in good health. At admission, he reported no palpitations, shortness of breath, fever, cough, or sputum production. His appetite, sleep, and bowel habits were normal, with no significant weight change. The physical examination revealed a soft abdomen with no muscle guarding, tenderness, or rebound tenderness. No hepatosplenomegaly was palpable. Bilateral lumbar curves were symmetrical. No tenderness was noted at the costovertebral angles or costovertebral junctions. Renal percussion was non-tender bilaterally. No tenderness was present along the ureteral courses. No elevation or tenderness was noted in the suprapubic bladder region. Vital signs: Temperature 36.4 °C, heart rate 85 bpm, respiratory rate 16 bpm, blood pressure 140/85 mmHg, height 174 cm, weight 65 kg. Laboratory tests are within normal limits. Imaging findings: CT with triple-phase contrast enhancement of the kidneys showed normal size and morphology bilaterally. Abnormal enhancement was observed in the left renal parenchyma; An outwardly protruding nodular lesion measuring approximately 1.6 cm was noted at the margin of the right renal upper pole, demonstrating relatively uniform enhancement. No dilatation was observed in the bilateral renal pelvis or ureters. Imaging findings suggested the right renal upper pole nodule represented a hypovascular, space-occupying lesion (**Figure 1**). Subsequent MRI further corroborated these findings, demonstrating no significant enhancement and confirming the lesion’s poor blood supply (**Figure 2**). Both the ipsilateral and contralateral adrenal glands were visualized on CT and MRI. They appeared normal in size, morphology, and enhancement pattern. They were in their typical anatomical positions without any discernible anatomical connection or continuity with the intrarenal nodule at the right upper pole. Based on clinical presentation and imaging features, a preliminary diagnosis of right renal cell carcinoma was considered. Despite the lesion’s poor vascular supply, given the high clinical suspicion of malignancy and the tumor’s exophytic nature making it suitable for nephron-sparing surgery, we proceeded directly with laparoscopic partial nephrectomy. This approach was preferred over preoperative biopsy because it combined diagnostic and therapeutic objectives, avoiding potential risks associated with biopsy, such as bleeding, needle-track implantation, or inconclusive diagnosis, while simultaneously providing curative treatment for a suspected malignant lesion. Laparoscopic partial nephrectomy was performed. During laparoscopic exploration, the lesion appeared as a well-circumscribed, exophytic nodule protruding from the upper pole of the right kidney, with no clear plane of separation from the surrounding renal parenchyma. The cystic nature of the lesion was evident upon gentle manipulation. Gross examination of the resected specimen revealed a cystic mass measuring 2.5 cm × 1.2 cm × 0.6 cm, with a smooth, thin wall and no significant internal content. The lesion was embedded within renal tissue, consistent with an intraparenchymal location. Postoperative pathology revealed a cystic wall-like tissue mass measuring approximately 2.5 cm × 1.2 cm × 0.6 cm with a wall thickness of 0.1 cm, containing minimal renal tissue and no visible contents upon dissection. Pathology diagnosed ectopic adrenal tissue within the kidney with pseudocyst formation (**Figure 3**). Immunohistochemistry results showed: Ki-67 (+<1%), TFE3 (±), Vimentin (+), CD10 (–), CK-pan (-), CK7 (-), PAX-8 (-), Carbonic Anhydrase IX (-), Inhibin (+), Syn (+) (**Figure 4**). These immunohistochemical findings were instrumental in confirming the adrenal origin of the tissue and ruling out renal cell carcinoma. The patient recovered well postoperatively without complications and was discharged on the third postoperative day. At the one-year telephone follow-up, the patient reported no subjective discomfort and showed no signs of recurrence of ectopic adrenal tissue.

## Discussion

This report describes a rare case of intrarenal ectopic adrenal tissue with pseudocyst formation in a 46-year-old male who presented with painless gross hematuria. Preoperative imaging revealed a small, exophytic, hypovascular nodule at the right upper renal pole, leading to a strong suspicion of renal cell carcinoma (RCC) and subsequent laparoscopic partial nephrectomy. Final pathology, supported by immunohistochemistry, confirmed the diagnosis of ectopic adrenal tissue with pseudocyst formation, thereby highlighting a challenging benign mimic of RCC (5).

The preoperative misdiagnosis in our case stemmed from substantial overlap in both clinical presentation and imaging features between ectopic adrenal tissue and RCC. Painless gross hematuria is a hallmark clinical symptom of RCC. In contrast, it is an exceedingly uncommon presentation for ectopic adrenal tissue, which is typically discovered incidentally without associated symptoms (11). This symptomatic presentation significantly heightened the suspicion of malignancy. Radiologically, the lesion appeared as a well-defined, exophytic cortical nodule with minimal contrast enhancement on both CT and MRI. While such hypovascularity is atypical for conventional clear cell RCC, it aligns with the imaging profile of papillary RCC or other hypovascular renal neoplasms (21, 22). Moreover, the intraparenchymal location and lack of a clear cleavage plane from the renal cortex further obscured the distinction from a primary renal tumor. These overlapping features underscore why ectopic adrenal tissue, especially when cystic or pseudo-cystic, is prone to misinterpretation as RCC in routine clinical practice (5, 19, 23).

The clinical implications of misdiagnosing ectopic adrenal tissue as RCC are significant and multifaceted. Firstly, it may lead to unnecessary and potentially more extensive surgical intervention. While a small, exophytic RCC might be managed with partial nephrectomy as performed in our case, a larger or centrally located suspected RCC could prompt a radical nephrectomy, resulting in the irreversible loss of a greater portion of, or the entire, kidney. This carries increased surgical risk, longer recovery time, and a higher long-term risk of chronic kidney disease. Secondly, a misdiagnosis of RCC could subject the patient to unnecessary adjuvant therapies, such as targeted therapy or immunotherapy, which are associated with significant side effects, financial toxicity, and no benefit for a benign condition. Thirdly, the psychological burden on a patient diagnosed with cancer, including anxiety about prognosis, recurrence, and metastasis, is profound and unjustified when the lesion is benign. Finally, from a surveillance perspective, a patient with a history of RCC would typically undergo prolonged, intensive, and costly radiological follow-up protocols, which are unnecessary for benign ectopic adrenal tissue. Therefore, enhancing preoperative diagnostic accuracy for such mimicking lesions is not merely an academic exercise but a crucial step in preventing overtreatment, preserving renal function, avoiding treatment-related morbidity, and alleviating patient distress.

Definitive distinction between ectopic adrenal tissue and RCC relies on histopathology and immunohistochemistry. Histologically, ectopic adrenal tissue typically demonstrates benign adrenal cortical architecture with lipid-rich cells, lacking the nuclear atypia, necrosis, or infiltrative growth seen in RCC (3, 23). In our case, the tissue was arranged in a cystic wall-like structure without malignant features. Immunohistochemically, ectopic adrenal tissue expresses adrenal cortical markers, such as Inhibin, Synaptophysin, and melan-A, while being negative for renal lineage markers, including PAX-8, CD10, and Carbonic Anhydrase IX (CA9) (5, 24, 25). In contrast, RCC exhibits the opposite profile (24, 26). The immunohistochemical profile in our case—specifically, the positivity for adrenal markers (Inhibin, Synaptophysin) coupled with the negativity for renal markers (PAX-8, CD10, CA9)—provides definitive evidence of adrenal cortical differentiation and effectively rules out RCC. This highlights the crucial role of immunohistochemistry in resolving diagnostically challenging cases, particularly when biopsy material is limited or imaging results are inconclusive.

Hematuria is an uncommon presentation for ectopic adrenal tissue and more typically suggests urothelial or renal parenchymal pathology. In our patient, detailed intraoperative inspection and histopathological examination did not reveal evidence of mucosal erosion, cyst rupture, active inflammation, or vascular injury that could directly explain the bleeding. The pseudocyst wall was intact, and no significant hemorrhagic or inflammatory changes were identified. While it is plausible that the intraparenchymal cystic lesion exerted a mass effect or transient irritative influence on the adjacent collecting system, the hematuria may also have been incidental or related to a minor, self-limited event not captured on imaging or histology. This case emphasizes that hematuria should not be considered a typical feature of ectopic adrenal tissue (11). Its presence should instead prompt a thorough investigation for more common causes, including renal malignancy, to avoid anchoring bias.

The approach to managing intrarenal ectopic adrenal tissue is informed by its diagnostic difficulty and its low but present risk of subsequent hyperplasia or malignancy (5, 15). For lesions with indeterminate imaging and significant clinical concern for malignancy, proceeding to surgical resection resolves the diagnostic uncertainty. It serves as the primary treatment (18). It provides definitive histopathological confirmation and eliminates the lesion, thereby addressing any associated symptoms and mitigating any long-term risk. This case reinforces several key lessons for improving preoperative diagnosis: Intrarenal ectopic adrenal tissue with cystic change should be considered in the differential diagnosis of small, hypovascular, exophytic renal cortical nodules, particularly at the upper pole (27–30).In cases with atypical imaging or symptomatic presentation, preoperative biopsy may be considered to avoid unnecessary surgery, although its diagnostic accuracy and risk profile must be carefully weighed. Multidisciplinary correlation among radiologists, urologists, and pathologists is essential to heighten awareness of this rare entity and to optimize patient-specific management strategies.

As a single-case report, our findings lack generalizability. The diagnostic challenge presented here underscores the reliance on postoperative pathology for definitive diagnosis. Future multicenter studies and advanced imaging research are needed to better characterize this entity and to develop more reliable preoperative discriminators.

## Conclusion

Intrarenal ectopic adrenal glands with pseudocyst formation represent an important, albeit rare, benign mimic of RCC. Although preoperative diagnosis poses significant challenges, this entity should be included in the differential diagnosis for small, poorly perfused masses located at the renal cortex margin with a protruding growth pattern, particularly considering occasional case reports describing similar findings. Enhancing awareness of this lesion and carefully evaluating the role of preoperative biopsy in specific cases are crucial for developing individualized treatment strategies. Ultimately, laparoscopic partial nephrectomy remains a safe and effective option for managing such diagnostic uncertain lesions, serving as an intervention with both diagnostic and therapeutic value.

## Data Availability

The original contributions presented in the study are included in the article/supplementary material. Further inquiries can be directed to the corresponding author.

## References

[1] Tarçın G Ercan O . Emergence of ectopic adrenal tissues-what are the probable mechanisms? J Clin Res Pediatr Endocrinol. (2022) 14:258–66. doi: 10.4274/jcrpe.galenos.2021.2021.0148, PMID: 34569220 PMC9422908

[2] Lu D Yu N Ma X Zhang J Guo X . An ectopic adrenocortical adenoma in renal hilum presenting with Cushing’s syndrome: A case report and literature review. Med (Baltimore). (2018) 97:e13322. doi: 10.1097/MD.0000000000013322, PMID: 30557981 PMC6319990

[3] Liu Y Jiang YF Wang YL Cao HY Wang L Xu HT . Ectopic adrenocortical adenoma in the renal hilum: a case report and literature review. Diagn Pathol. (2016) 11:40. doi: 10.1186/s13000-016-0490-6, PMID: 27094262 PMC4837621

[4] Al-Janabi MH Salamah D Suleiman M Mansour M Salloum R . Ectopic adrenal tissue in the mesosalpinx of an older female: the fourth case report in the literature. Oxf Med Case Rep. (2024) 2024:omae024. doi: 10.1093/omcr/omae024, PMID: 38680768 PMC11049563

[5] Telli E Yaprak Bayrak B Teke K Akdaş EM Kara Ö . Intrarenal ectopic adrenal tissue mimics renal cell carcinoma in the kidney: A clinicopathological diagnostic challenge. Arch Esp Urol. (2025) 78:109–12. doi: 10.56434/j.arch.esp.urol.20257801.14, PMID: 39943641

[6] Singh Y Bharti JN Vishnoi JR . Serous cystadenoma with ectopic adrenal cell rest of ovary: A rare case report. J Midlife Health. (2022) 13:325–7. doi: 10.4103/jmh.jmh_156_22, PMID: 37324793 PMC10266566

[7] Ghattas S Maalouf H Rahban H Nakhleh G El Rassi Z El Asmar A . A rare case of ectopic adrenal gland in an adult inguinal cord lipoma: Case report and literature review. Int J Surg Case Rep. (2024) 116:109390. doi: 10.1016/j.ijscr.2024.109390, PMID: 38377897 PMC10943995

[8] Permana H Darmawan G Ritonga E Kusumawati M Miftahurachman M Soetedjo NN . An interesting case of hepatic adrenocortical carcinoma. Acta Med Indones. (2018) 50:257–9., PMID: 30333277

[9] Al-Janabi MH Allan A Salloum R . Incidental ectopic adrenal cortical tissue in the descending mesocolon in an elderly female: a first case report in the literature. J Surg Case Rep. (2023) 2023:rjad067. doi: 10.1093/jscr/rjad067, PMID: 36846835 PMC9950716

[10] Ho L Policarpio-Nicolas MLC . Ectopic adrenal cortical tissue presenting as gastrohepatic ligament “lymph node” diagnosed by fine-needle aspiration: A case report. Diagn Cytopathol. (2023) 51:391–4. doi: 10.1002/dc.25142, PMID: 37128139

[11] Sensu S Keser SH Gurbuz Y Barisik NO Gul AE . Clinicopathological evaluation of 15 ectopic adrenal tissues. Arch Iran Med. (2021) 24:301–5. doi: 10.34172/aim.2021.42, PMID: 34196190

[12] Gaffoor N Shetty A Vidhya V Nikhil PV Mysorekar V . Bilateral adrenal cortical rests: An interesting innocuous intruder in the fallopian tubes. Indian J Pathol Microbiol. (2025) 68:402–4. doi: 10.4103/ijpm.ijpm_976_23, PMID: 38727413

[13] Şahin Ç Taylan E Akdemir A Zekioglu O Seyidova P Ergenoglu AM . Ovarian serous cystadenoma with ectopic adrenal tissue in a 65-year-old patient: A case report. Int J Surg Case Rep. (2017) 33:89–91. doi: 10.1016/j.ijscr.2017.02.045, PMID: 28285211 PMC5350495

[14] Hafiz B Alturkistani F . Adrenal cortical rests in the fallopian tube: A case report and review of the literature. Cureus. (2022) 14:e27649. doi: 10.7759/cureus.27649, PMID: 36072218 PMC9439637

[15] Saygin I Cakir E Ercin ME Eyüboğlu I . Incidental retroperitoneal oncocytoma (Ectopic oncocytic adrenocortical adenoma): Case report and review of the literature. Indian J Pathol Microbiol. (2019) 62:132–5. doi: 10.4103/IJPM.IJPM_58_18, PMID: 30706878

[16] Wahbi S Cherkaoui SB Aynaou H Salhi H El Ouahabi H . Pheochromocytoma in an ectopic adrenal gland. Cureus. (2023) 15:e40068. doi: 10.7759/cureus.40068, PMID: 37425549 PMC10326456

[17] Ohsugi H Takizawa N Kinoshita H Matsuda T . Pheochromocytoma arising from an ectopic adrenal tissue in multiple endocrine neoplasia type 2A. Endocrinol Diabetes Metab Case Rep. (2019) 2019:19-0073. doi: 10.1530/EDM-19-0073, PMID: 31610522 PMC6790906

[18] Desai NS Dalvi A Oak S Kothari P Gupta A Deshmukh S . Ectopic adrenal tissue in an inguinal hernia sac: A case report. Urol Case Rep. (2025) 62:103145. doi: 10.1016/j.eucr.2025.103145, PMID: 40860471 PMC12374199

[19] Park WY Seo HI Choi KU Kim A Kim YK Lee SJ . Three cases of adrenocortical tumors mistaken for hepatocellular carcinomas/diagnostic pitfalls and differential diagnosis. Ann Diagn Pathol. (2017) 31:9–13. doi: 10.1016/j.anndiagpath.2017.05.016, PMID: 29146062

[20] Azari-Yam A Khorvash R . Incidental findings of ectopic adrenal cortical tissue in the umbilical hernia sac and uterine wall: report of two cases in adult females. J Surg Case Rep. (2025) 2025:rjaf316. doi: 10.1093/jscr/rjaf316, PMID: 40395740 PMC12089982

[21] Liang X Xue C Huang X Wei J Zhou J . Value of energy spectrum CT parameters in the differential diagnosis of high-grade clear cell renal cell carcinoma and type II papillary renal cell carcinoma. Acta Radiol. (2022) 63:545–52. doi: 10.1177/02841851211002830, PMID: 33779302

[22] Liang RX Wang H Zhang HP Ye Q Zhang Y Zheng MJ . The value of real-time contrast-enhanced ultrasound combined with CT enhancement in the differentiation of subtypes of renal cell carcinoma. Urol Oncol. (2021) 39:837.e19–.e28. doi: 10.1016/j.urolonc.2021.09.004, PMID: 34654644

[23] De Marchi D Tafuri A Mantica G Shakir A Scarfò F Passaretti G . Ectopic adrenal tissue in the kidney: A systematic review. Arch Ital Urol Androl. (2021) 93:481–8. doi: 10.4081/aiua.2021.4.481, PMID: 34933527

[24] Jorge S Capelo JL LaFramboise W Satturwar S Korentzelos D Bastacky S . Absolute quantitative proteomics using the total protein approach to identify novel clinical immunohistochemical markers in renal neoplasms. BMC Med. (2021) 19:196. doi: 10.1186/s12916-021-02071-9, PMID: 34482820 PMC8420025

[25] Wang X Ng CS Yin W Liang L . Application of TFE3 immunophenotypic and TFE3 mRNA expressions in diagnosis and prognostication of adrenal cortical neoplasms and distinction from kidney tumors. Appl Immunohistochem Mol Morphol. (2023) 31:9–16. doi: 10.1097/PAI.0000000000001090, PMID: 36476598

[26] Ortiz-Rey JA Fachal C Juaneda-Magdalena L Muñoz-Martín M Repáraz-Andrade A Teijeira S . Clear cell clusters in the kidney: a rare finding that should not be misdiagnosed as renal cell carcinoma. Virchows Arch. (2021) 479:57–67. doi: 10.1007/s00428-021-03018-4, PMID: 33447899

[27] Miller C Raza SJ Davaro E Cao G Hamilton Z . Adrenal-renal fusion with adrenal cortical adenoma and ectopic adrenal tissue, presenting as suspected renal mass: A case report. Curr Urol. (2020) 14:163–5. doi: 10.1159/000499244, PMID: 33224009 PMC7659413

[28] Liu T Lv R Hu X Li K Ren Q Zhang Y . Case report: An ectopic adrenocortical adenoma in the renal sinus. Front Oncol. (2022) 12:934862. doi: 10.3389/fonc.2022.934862, PMID: 35965562 PMC9366061

[29] Endo M Fujii H Fujita A Takayama T Matsubara D Kikuchi T . Ectopic adrenocortical adenoma in the renal hilum mimicking a renal cell carcinoma. Radiol Case Rep. (2022) 17:619–22. doi: 10.1016/j.radcr.2021.10.038, PMID: 34987692 PMC8703184

[30] Lin D Qin J Xing J . Ectopic adrenocortical nodular hyperplasia mimicking an upper-pole renal-cell carcinoma: A case report. Asian J Surg. (2023) 46:2156–7. doi: 10.1016/j.asjsur.2022.11.092, PMID: 36456437

